# 3D Echo systematically underestimates right ventricular volumes compared to cardiovascular magnetic resonance in adult congenital heart disease patients with moderate or severe RV dilatation

**DOI:** 10.1186/1532-429X-13-78

**Published:** 2011-12-08

**Authors:** Andrew M Crean, Neil Maredia, George Ballard, Ravi Menezes, Gill Wharton, Jan Forster, John P Greenwood, John D Thomson

**Affiliations:** 1 Division of Medicine (Cardiology), Peter Munk Cardiac Centre, Toronto General Hospital, Toronto, Ontario, Canada; 2 Division of Adult and Pediatric Cardiology, Leeds General Infirmary, Leeds, UK; 3 Department of Medical Imaging, University of Toronto, Toronto, Ontario, Canada; 4 Academic Unit of Cardiovascular Medicine, Leeds General Infirmary, Leeds, UK

## Abstract

**Background:**

Three dimensional echo is a relatively new technique which may offer a rapid alternative for the examination of the right heart. However its role in patients with non-standard ventricular size or anatomy is unclear. This study compared volumetric measurements of the right ventricle in 25 patients with adult congenital heart disease using both cardiovascular magnetic resonance (CMR) and three dimensional echocardiography.

**Methods:**

Patients were grouped by diagnosis into those expected to have normal or near-normal RV size (patients with repaired coarctation of the aorta) and patients expected to have moderate or worse RV enlargement (patients with repaired tetralogy of Fallot or transposition of the great arteries). Right ventricular end diastolic volume, end systolic volume and ejection fraction were compared using both methods with CMR regarded as the reference standard

**Results:**

Bland-Altman analysis of the 25 patients demonstrated that for both RV EDV and RV ESV, there was a significant and systematic under-estimation of volume by 3D echo compared to CMR. This bias led to a mean underestimation of RV EDV by -34% (95%CI: -91% to + 23%). The degree of underestimation was more marked for RV ESV with a bias of -42% (95%CI: -117% to + 32%). There was also a tendency to overestimate RV EF by 3D echo with a bias of approximately 13% (95% CI -52% to +27%).

**Conclusions:**

Statistically significant and clinically meaningful differences in volumetric measurements were observed between the two techniques. Three dimensional echocardiography does not appear ready for routine clinical use in RV assessment in congenital heart disease patients with more than mild RV dilatation at the current time.

## Background

Monitoring of serial change in right ventricular (RV) size and function is of fundamental importance for physicians caring for patients with paediatric and adult congenital heart disease (ACHD) [1,2]. Two dimensional echocardiography is used extensively for this purpose but is inadequate for assessment of the complex geometry of the right ventricle without mathematical modelling [3]. Cardiovascular magnetic resonance (CMR) has become the reference standard for the measurement of right ventricular size, geometry and function. CMR benefits from excellent reproducibility of volumetric measurements of both ventricles and does not depend on a suitable acoustic window. However availability of CMR is limited and it can not be performed in the outpatient clinic or at the bedside.

Recently, three dimensional echocardiographic (3D echo) techniques have been introduced which are capable of acquiring a real time volumetric data set using ordinary commercially available echocardiography systems. Such data can be rapidly collected at the bedside and can be processed off-line in a similar manner to CMR data. If accurate and reproducible, this modality could simplify serial data collection in patients known to be at risk of the deleterious effects of right heart dilation and dysfunction. The purpose of this study was to report our initial experience of 3D trans-thoracic echo as a possible alternative to CMR in an ACHD population with a range of right ventricular volumes and functional abnormalities.

## Methods

### Subjects

Patients were recruited in a prospective, consecutive manner from the Adult Congenital Heart Disease out-patient program at our institution. Patients were eligible if they had undergone a CMR examination in the preceding 12 months, or if the responsible physician indicated that CMR exam would be performed in the subsequent 6 months. Recruitment was restricted to 3 groups; those with previously repaired Tetralogy of Fallot (ToF) in infancy or childhood (without subsequent pulmonary valve replacement); those with a right ventricle in the systemic position (e.g., Senning, Mustard or congenitally corrected transposition patients) and those with repaired coarctation (CoA) of the aorta. Patients within the first 2 groups were expected to have a range of RV dilatation and dysfunction, and for the purpose of analysis were treated as a single group. The coarctation group were included as an internal control arm with the expectation that right ventricular size and function would be normal or near-normal. The study was approved by the Institutional Review Board and all subjects gave written informed consent.

### 3D echocardiography

**Figure 1:**
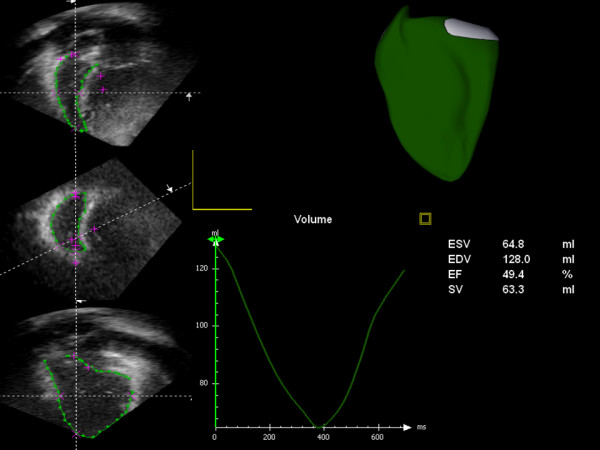
**Off line processing of 3D echo data**. Reconstructions are performed to generate images in 4 chamber, short axis and right ventricular inflow-outflow views. Contours are applied in all 3 planes at end systole and diastole and propagated in a semi-automatic fashion across all acquired time points. This allows generation of a time-resolved volume rendered image of the right ventricle (see Additional File 1: supplementary video file) and also a time volume curve from which functional data are obtained.

All patients underwent a full 2D trans-thoracic echocardiogram with particular focus on the right ventricle according to the recommendations of the American Society of Echocardiography [4]. All images were acquired by 2 British Society of Echocardiography-accredited sonographers with over 40 years of echocardiography experience between them (JF, GW). The standard 2D clinical examination was followed by additional full volume 3D image data acquisition (Philips IE33, Phillips Medical Systems, The Netherlands) by one of two experienced cardiac sonographers. Three dimensional image acquisition was performed from modified standard views (most often apical 4 chamber) in order to maximize visualization of the RV. The 3D echo datasets were analysed offline on a dedicated workstation (TomTec v1.2, TomTec Imaging Systems, Germany). Two dimensional multiplanar reconstructions of the RV were semi-automatically generated with manual correction to produce data sets in the short axis, four chamber and RV inflow-outflow orientation. End diastole and end-systole were selected manually by review of individual image phases and contouring was performed by semi-automatic border detection after manual placement of key seed points to define the RV apex and tricuspid annular plane (Figure 1). Resulting contours were checked for accuracy and corrected as necessary. Precise details of this process with the TomTec software package have been published recently [5].

### Cardiovascular MR

CMR examinations were performed on a Philips 1.5 T Intera magnet (Philips Medical Systems, The Netherlands). Steady state free precession (SSFP) images were acquired in the short axis plane from the atrioventricular groove to the cardiac apex. Cine acquisitions were performed with vectorcardiographic ECG gating over 6-10 heartbeats at held end-inspiration. Technical parameters were: slice thickness 8 mm no gap, 25 cardiac phases, TR 3.2 ms, TE 1.6 ms, FoV 320 × 320 cm, reconstruction matrix 256 × 256 cm. CMR studies were analysed offline using QMass V6.1 software (Medis, The Netherlands). ***All RV contours were performed on the short axis cine stack from the pulmonary valve to the RV apex, with trabeculation assigned to the blood pool ***[6]. Selection of end systole/diastole was performed manually by visual assessment of smallest/largest RV cavity size in each cardiac phase. RV volumes were calculated by the method of summated discs according to Simpson's rule [7].

### Statistics

Data are presented as mean (± SD). Descriptive statistics were used for normally distributed data. Non parametric statistics were employed to compare differences in volumes and function where appropriate. Fishers exact test was used for the comparison of proportions. Intra- and inter- observer analysis were tested for both techniques after an interval of 8 weeks by the original readers and a further reader analyzing a random selection of 50% of the cases from each imaging modality. Intra-class correlation coefficients were calculated to rate both inter and intra-observer variability in measurements of RV size and function. The method of Bland and Altman was used for assessment of systematic bias between methods of measurement [8]. P < 0.05 was taken to indicate statistical significance.

## Results

Twenty nine patients were recruited to the study over a 12 month period. Three patients failed to complete the study due to CMR-related claustrophobia and in 1 patient 3D echo was unsuccessful due to a combination of difficult echo window and extreme cardiac rotation. The study group was therefore comprised of 25 patients who underwent both 3D echo and CMR within a mean of 12 weeks of each other. Seven patients had a diagnosis of CoA, 14, had ToF and 4 had a diagnosis of complete transposition of the great arteries (TGA) palliated with either a Mustard or Senning procedure. The mean age was not significantly different between the 2 groups (CoA 26 yrs vs ToF/TGA 27 yrs p = ns). Detailed patient characteristics are given in Table 1. There were no significant differences between the 2 groups for patient body surface area or time between CMR and 3D echo examinations.

**Table 1:** Patient demographics and cardiovascular pathology of the study group

Variable	Normal RV group	Abnormal RV group	P value
	CoA	ToF/TGA	
**Age (years)**	26 (6.8)	27 (5.2)	ns
**Number (male)**	7 (5)	18 (7)	ns
**BSA**	1.76 (0.18)	1.71 (0.22)	ns
**Days between CMR & 3D echo**	125 (145)	120 (149)	ns

Data presented as mean (SD) or absolute values. CoA, aortic coarctation; ToF, Tetralogy of Fallot. BSA, body surface area.

**Figure 2:**
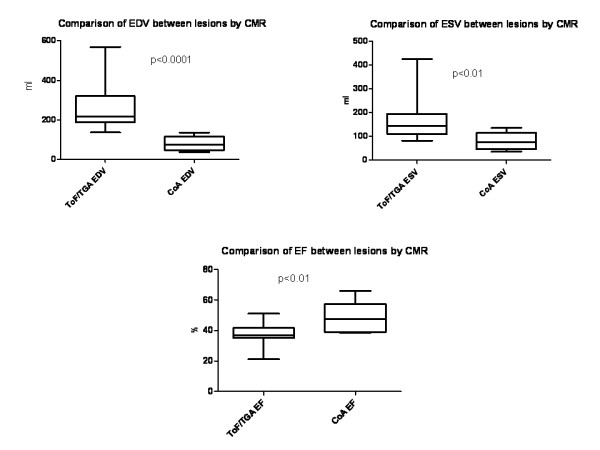
**a-c - Comparative RV volumes & function by modality**. Comparison of RV volumes and function between the mixed lesion (ToF and TGA) group and the CoA group. Boxes represent median and inter-quartile ranges and whiskers are the 95%CI.

Using CMR as the reference standard, there was a clear difference in RV size between the CoA group and the mixed lesion group (ToF and TGA). As expected, patients with a diagnosis of ToF or palliated TGA had significantly larger right ventricular end diastolic and end systolic volumes (EDV and ESV) than the patients with repaired CoA; the RV ejection fraction was also significantly lower in the former group (Figure 2).

**Figure 3:**
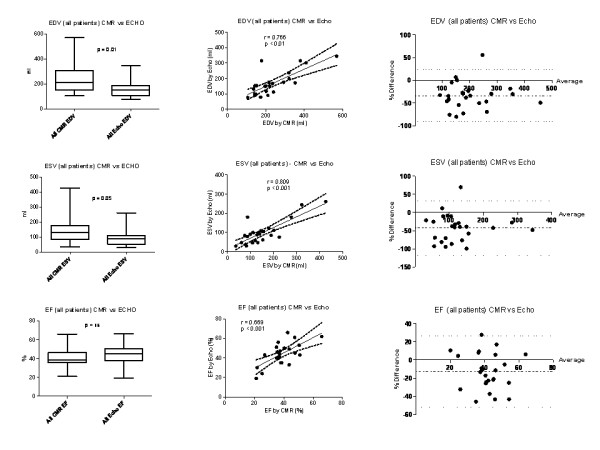
**a-i - Systematic differences between modalities for all patients combined**. Groups comparison, correlation and Bland Altman analysis of difference in RV volumes and function as measured by both CMR and 3D echocardiography. Boxes represent median and inter-quartile ranges and whiskers are the 95%CI. Bland Altman plots demonstrate mean bias (dot-dash line) and 95% CI (dotted lines).

Compared to CMR, 3D echo significantly underestimated volumes in the 25 patients as a whole, although the difference in measured RV ejection fraction (EF) was not significant (Figure 3). Mean RV end diastolic volumes were significantly greater when measured by CMR compared to 3D echo (236 (107) ml vs. 169 (78) ml; p < 0.01). Mean RV end systolic volumes were also significantly greater by CMR compared to 3D echo (146 (85 ml) vs. 98 (60) ml; p < 0.05). However, mean RV EF was not statistically different between the 2 modalities (40% (10%) vs. 44% (11%) for CMR and 3D echo respectively; p = 0.09).

Bland-Altman analysis of the 25 patients demonstrated that for both RV EDV and RV ESV, there was a significant and systematic under-estimation of volume by 3D echo compared to CMR (Figure 3). This bias led to a mean underestimation of RV EDV by -34% (95%CI: -91% to + 23%). The degree of underestimation was more marked for RV ESV with a bias of -42% (95%CI: -117% to + 32%). There was also a tendency to overestimate RV EF by 3D echo with a bias of approximately 13% (95% CI -52% to +27%).

When patient data were examined by disease grouping, a different pattern was seen with respect to volumetric measurements (Table 2). Large differences were observed between volume measurements in the ToF and TGA patients with a mean underestimation by 3D echo compared to CMR in RV EDV of -80 ml (bias -36%; 95%CI: -99 to +27%). However 3D echo in the CoA group demonstrated a much smaller mean underestimation of only -35 ml (bias -27%; 95%CI: -12 to +67%). End systolic volume was also underestimated by 3D echo in the ToF and TGA patients (mean difference -64 ml; bias -45%; 95%CI: -123 to +32%), but, again, to a lesser extent in the CoA group (mean difference -21 ml; (bias -34%; 95%CI: -104 to +35%). RV ejection fraction by 3D echo was marginally more accurate in the CoA group than in the ToF/TGA group (mean bias +3.8% 95%CI: -17 to +25% versus +6% 95%CI: -10 to +22% respectively).

**Table 2:** Absolute quantitative RV volumes and ejection fraction according to imaging technique and disease grouping (n = 25).

	CMR EDV (ml)	3D Echo EDV (ml)	CMR ESV (ml)	3D Echo ESV (ml)	CMR EF%	3D Echo EF%
**All patients**	236 (107)	169 (78)	150 (88)	98 (60)	39 (10)	44 (11)

**Tetralogy/TGA**	270 (106)	190 (81)	178 (88)	114 (63)	36 (08)	42 (10)

**Coarctation**	148 (35)	114 (31)	79 (30)	58 (28)	48 (10)	52 (12)

Data presented as mean (SD).

Finally, both intra- and inter-observer variability were significantly lower for CMR than for 3D echo (Table 3).

**Table 3:** Intra class correlation coefficients for inter and intra-observer variability according to technique

	Intra-observer variability CMR	Intra-observer variability *3D echo*	Inter-observer variability CMR	Inter-observer variability *3D echo*
**EDV**	0.995 (0.988, 0.998)	0.741 (0.480, 0.881)	0.994 (0.974, 0.999)	0.668 (0.150, 0.898)

**ESV**	0.993 (0.984, 0.997)	0.668 (0.361, 0.844)	0.996 (0.980, 0.999)	0.639 (0.099, 0.888)

**SV**	0.934 (0.855, 0.970)	0.694 (0.384, 0.863)	0.981 (0.920, 0.996)	0.261 (-0.371, 0.728)

**EF**	0.936 (0.861, 0.971)	0.491 (0.108, 0.747)	0.949 (0.791, 0.988)	0.428 (-0.197, 0.805)

## Discussion

The right ventricle is a complex geometric structure which unlike the left ventricle does not benefit from relative symmetry around its long axis. There are numerous published techniques for echocardiographic measurement of RV function [9-13]. However, conventional 2 dimensional echo techniques commonly underestimate the true size of the adult right ventricle [3]. This is a particular problem in Adult Congenital Heart Disease (ACHD) populations, since many surgical and interventional procedures may be considered even in asymptomatic patients based on certain volumetric thresholds. Furthermore, patients with functionally impaired single or systemic right ventricles may develop heart failure syndromes once the EF drops below 35%, emphasizing the relevance of accurate EF-derived risk stratification [14]. Accurate knowledge of poor EF is crucial in this patient group since it results in closer monitoring and follow up, more aggressive medication and pacing strategies, and - where appropriate - earlier referral for transplant assessment.

Three dimensional echocardiography offers the promise of accurate measurement of the RV without the need for geometric assumptions. Three dimensional echo is not a new technique - both in vitro and animal studies have shown efficacy in the measurement of left ventricular structures [15,16]. Further studies have demonstrated the utility of the technique for the assessment of left ventricular structures in both children [17] and adults [18]. However the left ventricle is a geometrically less complex structure than the right ventricle and it is thus better served by the mathematical underpinnings of 3D echocardiography.

Although there are right ventricular data from 3D echo in the pediatric age range, there are only isolated studies in the literature which have attempted to validate 3D echo in adult populations with congenital heart disease [19,20]. Our study was a direct comparison of trans-thoracic 3D echo versus CMR as the reference standard for measurement of right ventricular end-diastolic and end-systolic volumes and derived ejection fraction. We deliberately included adult patients with repaired tetralogy of Fallot since the majority of these have had prior patch enlargement of the RV infundibulum with disruption of the pulmonary valve annulus resulting in severe pulmonary incompetence and progressive RV dilatation over time. The severity of RV dilatation in ToF patients in our study was significant though not extreme, with a mean EDV of 270 ml (corrected by body surface area to a mean RV EDV index of 153 ml/m^2^). One currently available retrospective study suggests a threshold of 180 ml/m^2 ^at which pulmonary valve replacement (PVR) should be considered [21]. In reality, there is considerable variation in threshold for surgical referral for PVR from center to center as well as on a patient to patient basis [22]. Nonetheless it is important that the selected measurement methodology should be accurate and reproducible within the range of moderate to severe RV dilatation.

The inclusion of several patients with transposition of the great arteries and Mustard/Senning repair was merited as these patients have the right ventricle located in the subaortic position. This inevitably leads to secondary hypertrophy and wall thickening, as well as atrio-ventricular valve regurgitation and secondary systemic ventricular enlargement. Serial assessment of the systemic ventricle in these patients is imperative as progressive decline is often an indication for more aggressive therapies including heart transplantation.

CMR has been shown to have excellent reproducibility for measurement of both LV and RV size and function [23], and this was again confirmed for the right ventricle in our study, despite the relative severity of dilatation. Our results with regard to the accuracy and reproducibility of 3D echo, however, are less positive than reported in the published literature.

Earlier work comparing RV volume measurement by both transthoracic and transesophageal 3D techniques have demonstrated excellent correlations between the 3D echo measurements of RV volume and function compared to both CMR and radionuclide ventriculography [24]. However, this patient population was more heterogeneous than ours and included only a single patient with pathology associated with RV dilatation (secundum ASD). Review of their data reveals that the mean (SD) RV EDV by CMR was only 109 (34) ml, *uncorrected for BSA*, with a maximum EDV of just 191 ml. This compares with an uncorrected mean (SD) and maximum in our series of 236 (107) ml, and 509 ml respectively. Despite the relatively small RV cavity sizes included, Nesser et al observed: "...on the Bland-Altman scattergram....a tendency for TEE-3D and TTE-3D to underestimate large RV volumes compared with MRI..." - as such their data are entirely concordant with our findings in much larger RVs.

Recently, Grewal et al examined 25 patients with tetralogy of Fallot using both CMR and 3D echo [19]. Although they demonstrated better correlations between the two modalities for RV volumes than in our own study, they nonetheless describe a systematic underestimation of both RV EDV and ESV by a maximum of up to 36%. As in our study and that of Nesser et al, the greatest discrepancy between the two techniques occurred in patients with larger right ventricles, particularly above a threshold of 250 ml for EDV.

Further comparable work was published recently by Arnould et al in an adult population suffering from assorted cardiomyopathies [25]. The mean RV EDV by CMR in that study was 171 (69) ml but this was severely underestimated by 3D echo at only 77 (42) ml. End systolic volume was also significantly underestimated by a mean of almost 60 ml (ESV CMR 105 (55) ml vs. ESV 3D echo 46 (32) ml).

Our results with 3D echo are similarly disappointing with respect to patients with right ventricular enlargement due to congenital heart disease. As we hypothesized, measurements made by CMR and 3D echo were comparable in the coarctation patient group who had normal RV size. However the patient group in whom 3D echo potentially has the most to offer, those with an enlarged RV (e.g. the ToF and TGA groups), demonstrated relatively poor comparison to CMR for volumetric indices. Furthermore 3D echo would have been falsely reassuring with respect to RV ejection fraction in a number of patients in this study.

The reasons for the limited accuracy and reproducibility of 3D echo are likely to be multi-factorial: the spatial resolution of 3D echo in full volume mode is substantially lower than that of the native 2D application; limited lateral spatial resolution results in poorly-defined 'fuzzy' endocardial borders in diastole, and creates genuine difficulty in separating endocardium from trabeculation at end-systole. This specific point was touched on in detail in a thoughtful editorial by Mor Avi et al who point out that a similar difference in recorded volume between the 2 techniques is also seen for the left ventricle *but that this difference may be substantially reduced if CMR-contouring is performed in such a manner as to exclude the trabeculation from the blood pool *(which is generally the opposite of current practice) [26]. In other words current conventions of contour drawing tend to favour high resolution techniques like CMR in comparisons against lower resolution methods [5]. As Mor Avi poignantly comments: 'the devil is in the boundary' [27].

Secondly, the right ventricle may be difficult even in a normal individual to fully encompass within a pyramidal volume as is required during 3D image acquisition. In many cases the available sector width is simply inadequate for the size of the ventricle, leading to poor or incomplete endocardial definition in the reconstructed views, with adverse effects on the accuracy of contour definition. Future technical developments will no doubt address the difficulties imposed by sector angle limitations. Thirdly, a proportion of patients may have difficult acoustic windows regardless of RV size. Not infrequently this is related to musculoskeletal abnormalities, such as pectus excavatum, which are often seen in patients with congenital heart disease.

Finally, a degree of selection bias undoubtedly plays a role in published data. We deliberately included all but one patient in our analysis; this patient was excluded as the 3D echo image quality was so poor that no attempt at contouring could be made. However one recent publication comparing manual versus automated border detection of 3D echo (versus CMR) in a similar population to ours documented *a 48% exclusion rate from data analysis *in 54 consecutive patients scanned, because of inadequate image quality [20]. The authors explain that they found, as we did, that difficulties in imaging the near field by 3D echo results in poor definition of the anterior free wall of the right ventricle with potential inaccuracies for extrapolation of the endocardial contour. Although the findings of these authors were undoubtedly improved by limiting their analysis to good quality data sets, the need to exclude half the recruited sample would preclude translation of this technique into routine clinical practice.

### Limitations

We acknowledge several limitations to our study. Firstly our sample size was relatively small (though not dissimilar to the published literature in this area) and in particular lacked power to clearly define the progressive limitations of 3D echo in reference to graded increases in RV end-diastolic volume. Secondly, although our patient recruitment was prospective and consecutive the 3D echo was not performed on the same day as the CMR study; however the mean time difference was only 12 weeks between studies in which time one would not expect any significant systematic change in RV cavity size in a stable outpatient population. Thirdly, since 3D echo currently acquires a 'pyramid' of data over multiple sequential heartbeats we did not attempt to recruit any patient who was not in sinus rhythm. This is also a relative limitation of CMR which generally requires a stable R-R interval for routine segmented cine imaging. Fourthly we acquired CMR images in end-inspiration rather than end expiration as is done more routinely. This is because 3D echo windows were generally superior at end-inspiration and we wished to match the two techniques as far as possible. Limited volunteer data suggests this entails a risk of 'over-sampling the RV' since the end inspiratory position of the diaphragm may vary by as much as 25% [28]. However the risk of scanning the same slice twice on sequential breatholds as a result is mainly a problem for scanning in the axial plane. We contoured the RV from the short axis plane in order to obviate this concern. Finally, we acknowledge a greater experience with both acquisition and post-processing of CMR images than 3D echo, which may have introduced a learning bias into our results. This is, however, the case whenever a new modality is compared to an existing reference standard and, as shown above, our results are not discordant with those seen in several studies of both adult congenital and acquired heart disease populations.

## Conclusions

This study provides further data describing a clinically important underestimation of RV volume by 3D echo, particularly in a patient subgroup with moderate or severe enlargement. We observed a systematic underestimation of both RV EDV and ESV when compared to CMR to an extent which has clinical relevance. The degree of underestimation may provide false reassurance to both patient and physician, and could have an adverse impact on appropriate clinical decision making.

3D echo is a new and rapidly evolving modality which may in the near future be able to offer the twin benefits of portability and comparable accuracy to CMR. Many factors contributing to the relative inaccuracy of 3D echo are technical issues that will be addressed by further research and product development. However current data suggest caution is warranted when using 3D echo as the principal imaging modality in patients with any greater than mild RV dilatation. Physicians caring for patients in whom management decisions are based upon possession of accurate volumetric data need to be aware of the current limitations in 3D echocardiography. Our data, and others, support the contention that CMR - for the time being at least - remains the standard of reference in this population.

## Competing interests

The authors declare that they have no competing interests.

## Authors' contributions

AMC, JT and JDT were responsible for study design. AMC, JDT and GB recruited patients for the study. GW and JF performed the 3D echo on all the patients included in the study. AMC and NM performed the CMR analysis. AMC and GB performed the 3D echo analysis. AMC and RM were responsible for the statistical analysis. All authors read and approved the final manuscript.

## Supplementary Material

Additional file 1 **Movie 1**. A typical example of the 4D model of the right ventricle derived from a trans thoracic 3D echocardiography study. Click here for file
